# An Item of Interest

**Published:** 1884-06

**Authors:** 


### AN ITEM OF INTEREST.

The editor of one dental journal raises his hands and eyes in holy horror over the enormities of tobacco smoking, but we cannot avoid the impression that he sometimes neglects the weightier matters of the law. He seems to be a kind of freebooter among the journals, and lays violent hands on whatever strikes his fancy. What would be thought of the morality of a man who, having borrowed (sometimes without permission) an implement from his neighbor, should immediately erase the owner's name, change its form a little, paint it over with another color, and then stoutly claim it for his own? But that is precisely what *Items of Interest* has many times done. Not, of course, with larcenous motives (we do not wish to be understood as charging willful turpitude), but with the object of fitting to its narrower pages articles that have cost their rightful owners both time and money, and which he has no legal or moral right thus to mutilate. Shall we particularize?

The March number contains such an one, which was the legitimate property of this journal. It is mutilated, and disguised, and then appropriated without so much as "thank you."

The same number contains an article which, according to the custom that obtains among journals, is claimed as original, and contributed directly to its pages by the author, Dr. C. S. Chittenden, of Hamilton, Ontario. It was read before the Michigan State Dental Society in 1862. The grossest injustice is done its author in now placing him before the profession as advancing views which he long ago discarded. If a journal can not present ideas that are less than a score of years old, we ask what is the excuse for its existence? In the name of the whole profession we protest against this reprinting without warrant of the antiquated ideas of men who have since outgrown them, and there are other and more pretentious journals that might not unprofitably ponder thereon. In this day of investigation progressive men are not immovably planted, like posts.

The April number contains an article purporting to be written by "Samuel Welchens, D. D. S., Rochester, N. Y.," and credit for it is given to the "District Dental Society of Rochester." There is not now, and never has been, a "Samuel Welchens, D. D. S.," in Rochester, nor has there ever been any such society as the "District Dental Society of Rochester." In 1875 Dr. Samuel Welchens, of Lancaster, Penn., contributed a paper for a meeting of the dentists of Western New York. Its subject was "The treatment of exposed pulps in filling teeth." It was read by the Secretary, and published in the *Missouri Dental Journal* nine years ago, and in the long ago deceased *Pennsylvania Journal of Dental Science*, of which Dr. Welchens was at that time the editor. It has been resurrected, revamped, divided, given a new head, and it now makes two fresh, original communications for *Items of Interest.* It was an excellent paper in its day, but has an ancient and fish-like smell as it now appears.

To show that this is not a mere typographical or unintentional error, the May number contains another instalment of the same thing, the same pretended author and society. The second article was taken from this journal without credit, and there is another from Dr. Brophy—but why go on to enumerate instances that are comparatively numberless? We respectfully request that in future, *Items of Interest* will obey the common journalistic law, and respect the property of others. We are quite willing to lend to it anything we have, but we must ask it to refrain from rubbing out the ownership name of the INDEPENDENT PRACTITIONER, from wantonly mutilating our property, and from claiming as its own that which we have bought and paid for.

